# Study on the mechanism of Dexmedetomidine’s effect on postoperative cognitive dysfunction in elderly people

**DOI:** 10.3389/fphys.2025.1508661

**Published:** 2025-03-12

**Authors:** Yuanbin Cai, Fan Yu, Wei Wu, Wurong Chen

**Affiliations:** ^1^ Department of Anesthesiology, Putuo District Central Hospital, Shanghai, China; ^2^ School of Exercise and Health, Shanghai University of Sport, Shanghai, China

**Keywords:** postoperative cognitive dysfunction, dexmedetomidine, elderly, mechanisms, effect

## Abstract

Postoperative cognitive dysfunction (POCD) is a common complication among elderly patients following surgical procedures, significantly impairing postoperative recovery and quality of life. The selection and dosage of intraoperative anaesthetic drugs are frequently implicated as contributing factors in the development of POCD. In recent years, dexmedetomidine (DEX), a novel α2-adrenoceptor agonist, has been increasingly utilized in surgical anaesthesia for elderly patients, showing potential as both a preventive and therapeutic agent for POCD. This paper provides a comprehensive review of current research on the mechanisms by which DEX affects POCD in the elderly. Additionally, it explores DEX’s mechanisms of action in the context of neuroprotection, anti-inflammation, antioxidative stress, and the regulation of apoptosis, autophagy, and analgesia. The objective is to provide reliable theoretical support and a reference point for the clinical application of DEX in POCD among the elderly, thereby promoting its broader use in clinical practice to improve outcomes and enhance quality of life.

## 1 Introduction

Anaesthetics are classified into two main categories: local and general anaesthesia, and they are widely employed as analgesic and sedative agents in modern clinical practice (Neuman et al., 2021). Anaesthesia typically involves the transient suppression of central and/or peripheral nervous system function through the administration of anaesthetics via various routes, including oral, inhalation, local, intravenous, or intramuscular methods. This results in skeletal muscle relaxation of the patient’s skeletal muscles and the reversible loss of consciousness and pain sensation at either the local or whole-body level (Kamel et al., 2022; Zhang et al., 2023). However, the administration of anaesthetics inevitably induces various hazards and side effects (Glannon, 2014). For instance, anaesthesia may result in long-term neurodevelopmental consequences in neonates, manifesting as neuronal developmental disorders (Ing et al., 2014; Lee et al., 2015). In adults, anaesthesia may lead to mild nausea and vomiting (Jenkins and Baker, 2003). Among the elderly, anaesthetics have been associated with adverse outcomes such as neurocognitive dysfunction and, in severe cases, mortality (Häusler et al., 2022; Koo and Ryu, 2020; Amare et al., 2019). Consequently, there is a growing need to develop effective anaesthetics with minimal side effects, particularly for elderly patients.

Postoperative cognitive dysfunction (POCD) is a central nervous system complication that frequently occurs after surgical procedures, particularly in elderly patients (Luo et al., 2019; Lin et al., 2020; Hua and Min, 2020). It is characterized by a gradual decline in cognitive abilities and memory, along with personality changes, psychiatric disorders, memory impairment, and inattentiveness (Li et al., 2022; Kotekar et al., 2018). Studies have shown that the prevalence of POCD in elderly patients ranges from 25.8% to 41.4% within 1 week of surgery (Shen et al., 2022). This condition results in prolonged hospitalization, increased economic burden, diminished quality of life, and even heightened mortality rates (Shao et al., 2019). The current understanding of POCD suggests that its occurrence is influenced by intrinsic factors, such as age, pre-existing cognitive impairment, and cerebrovascular disease, as well as extrinsic factors, including the type of surgery, anaesthetic agents used, and postoperative infection (Schenning and Deiner, 2015; Rosczyk et al., 2008; Czyż-Szypenbejl et al., 2019; Wang C. M. et al., 2021; Cao et al., 2019). Elderly individuals are particularly vulnerable to the effects of anaesthetic agents on the brain compared to middle-aged individuals. Furthermore, the choice of anaesthetic significantly affects the severity and duration of POCD (Vutskits and Xie, 2016).

Dexmedetomidine (DEX) is a novel α2-adrenoceptor (α2-AR) agonist with a binding ratio to α2: α1 adrenoceptors of approximately 1,620:1 (Zhou et al., 2022; Mahmoud and Mason, 2015; Weerink et al., 2017; Tishchenko and Dobrodeev, 2019; Wang et al., 2017). Studies have demonstrated that DEX reduces the incidence of POCD by protecting neuronal function (Wang D. et al., 2021; Prommer, 2011; Takada et al., 2002), while having minimal impact on postoperative cognition in patients over 60 years of age (Yang et al., 2023). In animal models, DEX has been shown to provide neuroprotection against brain injury in ischaemic-hypoxic neonatal rats, attenuate POCD in aged rats, and promote neurogenesis and cognitive recovery in mice following surgery (Fang et al., 2018; Wang W. X. et al., 2018). Evidence suggests that DEX may serve as a promising agent for intraoperative neuroprotection in elderly patients (Kallapur and Bhosale, 2012; Djaiani et al., 2016). Accordingly, this study aims to investigate the effects and mechanisms of DEX on the nervous system and to discuss its ameliorative effects of DEX on POCD, with the goal of providing a more robust theoretical foundation for the safe of clinical use of anaesthetics in elderly surgical patients.

## 2 Pharmacological effects of DEX

### 2.1 Sedative effects

The locus coeruleus, often referred to as the blue spot, is the brain region with the highest density of α2-AR and plays a key role in the regulation of wakefulness and sleep (Liang et al., 2021). DEX produces dose-dependent sedative, hypnotic, and anxiolytic effects by activating α2-AR in the locus coeruleus, initiating endogenous sleep mechanisms (Tasbihgou et al., 2021). Unlike other anaesthetic agents, which induce sedative-hypnotic effects through pharmacological pathways, DEX induces sedation comparable to natural sleep, allowing patients to be easily awakened by verbal or tactile stimuli without causing respiratory depression (Chima et al., 2022). In surgical patients undergoing craniofacial functional therapy, DEX has been shown to provide optimal sedation while enabling the completion function tests during arousal (Kallapur and Bhosale, 2012). The sedative effect of DEX depends on its plasma concentration. At concentrations of 0.2–0.3 ng/mL, DEX induces mild to moderate sedation, whereas at concentrations exceeding 1.9 ng/mL result in deep sedation, which can be deleterious to patients (Weerink et al., 2017).

### 2.2 Analgesic effects

DEX has demonstrated significant analgesic properties. In the brain, DEX binds to α2-AR in the locus coeruleus, inhibiting the transmission of pain signals. In the spinal cord, DEX activates α2-AR on the presynaptic membranes of posterior horn neurons and postsynaptic membranes of intermediate neurons. This activation opens K^+^ channels, facilitating K^+^ efflux while inhibiting Ca^2+^ influx. Consequently, cell membranes become hyperpolarized, blocking of the medulla oblongata-spinal cord conduction pathway and inhibiting the central transmission of pain signals (Bahari and Meftahi, 2019). Additionally, DEX exerts analgesic effects by stimulating peripheral nerve cells to release choline-like substances, thereby increasing the pain threshold (Doze et al., 1989). When combined with other analgesic agents, DEX demonstrates synergistic effects, allowing for dose reductions and minimizing associated adverse effects (Blaudszun et al., 2012; Venn et al., 1999; Lin et al., 2009; Kalaskar et al., 2021; Tsaousi et al., 2018; Wang X. et al., 2018). For example, Mueller et al. (2014) showed that combining DEX with morphine enhances both analgesic and sedative effects while significantly reducing the required dosage and side effects of morphine. Furthermore, DEX not only provides mild intrinsic analgesic effects but also potentiates the analgesic efficacy of opioids (Song et al., 2016; Mathai et al., 2019).

### 2.3 Anti-sympathetic effects

DEX has been shown to suppress the excitability of the sympathetic nervous system by activating α2-AR in the locus coeruleus of the brainstem. This activation inhibits norepinephrine release, reduces plasma catecholamine levels, and stabilises haemodynamics, providing anxiolytic, antidepressant, and neuroprotective effects (Mondardini et al., 2019). DEX’s anti-sympathetic action also attenuates the surgical stress response, significantly reducing plasma catecholamine and cytokine release, which mitigates the development of hypercoagulability during surgical procedures (Ramadhyani et al., 2010). Moreover, by enhancing parasympathetic activity and activating cholinergic anti-inflammatory pathways, DEX suppresses inflammatory responses in local tissues in the brain and various other organs during the perioperative period, thus affording organ protection (Bajwa and Kulshrestha, 2013). However, the reduction in sympathetic nervous tension is dose-dependent and can lead to bradycardia and hypotension, which are among the most common adverse effects of DEX (Schnabel et al., 2018; Yang and Gao, 2021). Therefore, careful monitoring is required, particularly in patients with hypovolaemia or pre-existing arrhythmias.

### 2.4 Attenuation of respiratory depression

DEX exerts a minimal effects on the respiratory function. It slightly reduces minute ventilation and causes a mild increase in arterial carbon dioxide partial pressure (PaCO_2_) during deep sedation. However, it does not suppress the body’s response to hypercapnia, making its respiratory effects similar to those observed during natural sleep under continuous infusion (Venn et al., 2000; Cui et al., 2020). Gao et al. (2020) demonstrated that DEX inhibits neuronal apoptosis through mitochondrial pathways, mediates the neuroprotective effects via the regulation of neurotoxin expression, and protects against hypoxia/reoxygenation-induced neuronal damage in rat models. Additionally, DEX has been shown to reduce the incidence of adverse respiratory events during procedures such as craniotomy or tracheal intubation, outperforming opioids and propofol in this regard (He et al., 2019; Nguyen and Nacpil, 2018).

## 3 The effects of DEX on postoperative cognitive dysfunction

### 3.1 Suppression of inflammatory response

Surgical procedures are recognised as significant contributors to the development of POCD (Peng et al., 2023; Zhong et al., 2024; Zhou et al., 2023). Surgery-induced peripheral blood inflammatory factors in peripheral blood can activate microglia, eliciting excessive immune responses (Olotu, 2020; Chu et al., 2018). This process leads to the release of large quantities of inflammatory mediators, which can disrupt neurotransmitter signalling in the hippocampus and cause reversible or irreversible brain tissue damage. Such disruptions may alter neuronal synapses, contributing to neurodegenerative processes and cognitive dysfunction (Podjaski et al., 2015; Hoogland et al., 2015; Gyoneva et al., 2014). Elderly patients are particularly vulnerable to POCD due to the activation of the peripheral immune system, which amplifies central nervous system responses (Chen et al., 2019; Ye et al., 2024). Numerous studies have demonstrated that DEX reduces hippocampal inflammation by downregulating the expression of tumour necrosis factor-α (TNF-α), interleukin-6 (IL-6), and interleukin-1β (IL-1β), thereby inhibiting neuronal apoptosis and protecting against POCD (Wang et al., 2022; Frakes et al., 2014). The nuclear factor kappa-B (NF-κB) pathway plays a pivotal role in neuroinflammation by regulating inflammatory mediators in microglia (Xu et al., 2013; Park et al., 2015). Sun et al. (2021) showed that DEX inhibits microglial activation and reduces inflammatory mediator release by suppressing the NF-κB pathway. Similarly, Zhou et al. (2020) demonstrated that DEX pretreatment ameliorates lipopolysaccharide (LPS)-induced cognitive deficits in aged mice by inhibiting the TLR4/NF-κB pathway in the hippocampus. These findings suggest that DEX exerts a protective effect against POCD through the reduction of inflammatory factor secretion.

### 3.2 Antioxidant stress

Oxidative stress, characterised by an imbalance between the production of reactive oxygen species (ROS) and reactive nitrogen species (RNS) production and the tissue’s antioxidant capacity, is a key contributor to POCD (Song et al., 2019; Gong et al., 2020; Liu H. et al., 2020; González-Domínguez et al., 2014). When cellular antioxidants fail to neutralise ROS effectively, oxidative damage to lipids, DNA, and proteins occurs, leading to neuronal injury and cognitive dysfunction (Kojima et al., 2013; Rodrigues et al., 2017; Lee et al., 2012). Mitochondria, the primary site of intracellular ROS production, play a crucial role in oxidative metabolism, cell survival, and apoptosis (Rizwan et al., 2020). Excessive ROS production reduces mitochondrial protein activity in neuronal cells, contributing to neurodegenerative diseases such as Alzheimer’s disease (AD) and Parkinson’s disease (PD) (Alavian et al., 2015).

The protective effect of DEX against POCD is closely associated with its antioxidative properties. DEX significantly inhibits ROS overproduction and cell apoptosis, thereby mitigating oxidative damage. Furthermore, the antioxidant effect of DEX ameliorates oxidative stress and apoptosis, improving POCD progression (Chen et al., 2018; Liu P. et al., 2020). Superoxide dismutase (SOD), a critical component of the antioxidant defence system, scavenges excess ROS and mitigates their adverse effects (Jiang et al., 2019). Studies have shown that DEX increases SOD levels in elderly patients following surgery, conferring neuroprotective effects on cognitive function (Xie et al., 2021). Thus, DEX exerts its protective effects on cognitive function by counteracting oxidative stress, thereby alleviating POCD-induced damage.

### 3.3 Inhibition of apoptosis

Apoptosis is a regulated and orderly form of cell death controlled by various genes. However, abnormalities in apoptotic processes can result in cell death triggered by specific injury factors and constitute a primary mechanism of delayed neural injury (Wnuk and Kajta, 2017). Increasing evidence indicates that hippocampal neuronal apoptosis is a major contributor to cognitive dysfunction in patients experiencing brain injury (Wang et al., 2024). Jevtovic-Todorovic et al. (2003) observed severe apoptosis and progressive, irreversible cognitive dysfunction in rats exposed to isoflurane, nitrous oxide, and midazolam for six hours. Similarly, propofol, a widely used intravenous anaesthetic, has been reported to disrupt synaptic plasticity, leading to neuronal apoptosis and damage, ultimately causing memory dysfunction (Lv et al., 2017). In contrast, DEX has demonstrated the ability to prevent POCD by inhibiting neuronal apoptosis (Si et al., 2016). Liu Y. J. et al. (2017) found that DEX significantly reduced propofol-induced neuronal apoptosis and neurocognitive dysfunction, providing neuroprotection. Furthermore, Sanders et al. (Sanders et al., 2010) reported that DEX mitigated hippocampal neuronal apoptosis and reduced brain injury in rats following isoflurane anaesthesia, thereby preventing POCD. These findings suggest that DEX protects against POCD by inhibiting neuronal apoptosis.

### 3.4 Regulation of cellular autophagy

Autophagy is a self-phagocytosis mechanism observed in eukaryotic cells, representing a biological process of cellular self-regulation. When cytoplasmic components or organelles are damaged or destroyed, autophagy is activated to protect the cell (Settembre and Ballabio, 2014). However, disruptions in autophagy that disturb cellular homeostasis can ultimately result in cell death (D'Arcy, 2019). Autophagy has been implicated in the pathogenesis of cognitive disorders. For instance, inhibition of autophagy has been shown to cause abnormal aggregation of α-synuclein, which exacerbates cognitive impairment in patients with AD (Ntsapi et al., 2018; Cuervo et al., 2004). In an animal study, Zhang et al. (2016) found that impaired autophagy following sevoflurane anaesthesia led to cognitive dysfunction in aged rats. Phosphorylation of adenosine monophosphate-activated protein kinase (AMPK), a key molecule in cellular autophagy, has been demonstrated to promote autophagy and alleviate cognitive deficits in aged rats with POCD (Niu et al., 2022).

DEX facilitates the initiation of autophagy-related signalling pathways by activating the AMPK signalling pathway (Wang J. et al., 2023). In addition, DEX promotes autophagy by inhibiting the mammalian target of rapamycin (mTOR) signalling pathway, a negative regulator of autophagy. Inhibition of mTOR enhances autophagic processes, thereby mitigating POCD (Yu et al., 2023). These findings suggest that DEX protects against POCD by promoting cellular autophagy.

### 3.5 Reduction of anaesthetic drugs

The combination of drugs represents an effective strategy for augmenting the protective effects of DEX against POCD. DEX, known for its sedative and hypnotic properties, can produce synergistic effects when combined with other sedative and analgesic drugs, thereby reducing the required doses of general anaesthetic agents (Liu X. et al., 2017). For instance, combining DEX with general anaesthesia not only reduces the dose of propofol but also decreases the incidence of adverse reactions associated with anaesthesia, such as numbness, convulsions, and vomiting (Liu et al., 2019). Furthermore, DEX has been shown to accelerate analgesic effects, lower visual analogue scale (VAS) scores, and improve early postoperative mini-mental state examination (MMSE) scores, thereby providing protection against POCD (Zhang T. et al., 2019). It can thus be inferred that DEX protects against POCD by reducing the dosage of other anaesthetic agents.

## 4 Role of multiple signalling pathways in POCD

### 4.1 DEX activates the PI3K/Akt signalling pathway to protect against POCD

Phosphatidylinositol 3-kinase (PI3K) is a critical intracellular signalling molecule activated by extracellular stimuli such as growth factors, cytokines, and hormones (Xie et al., 2019). Akt, also referred to as Protein Kinase B (PKB), is a key downstream effector of PI3K and undergoes phosphorylation under normal physiological conditions (Hers et al., 2011). Activation of Akt promotes cell survival by regulating apoptosis-related proteins, including caspase-9 and glycogen synthase kinase-3 (GSK-3) (Zhou et al., 2000). The PI3K/Akt signalling pathway is a well-established anti-apoptotic pathway with widespread expression across tissues, regulating processes such as growth, proliferation, differentiation, apoptosis, and metabolism (Zhang et al., 2022). The PI3K/Akt pathway also plays a role in inflammatory responses. Pro-inflammatory cytokines, including interleukin-1β (IL-1β), interleukin-37 (IL-37), and tumour necrosis factor-α (TNF-α), activate Akt and amplify the inflammatory response. This activation induces autophagy and apoptosis through the PI3K/Akt pathway, contributing to inflammation by influencing neutrophils and lymphocytes (Xue et al., 2015; Eräsalo et al., 2018).

Extensive evidence indicates that the PI3K/Akt signalling pathway is pivotal in the neuronal injury associated with surgical trauma, anaesthesia, and hypoxia, particularly in POCD (Qin et al., 2020). Xiao et al. (2018) demonstrated that DEX pretreatment enhanced PI3K/Akt activation in the hippocampus of juvenile rats, reduced propofol-induced neuronal apoptosis, alleviated long-term neurotoxicity, and improved spatial learning and memory. Similarly, Zhang et al. (2020) found that DEX attenuated neuronal apoptosis in rats with transient focal ischemia-reperfusion through the PI3K/Akt pathway, reducing brain injury. These findings suggest that DEX mitigates surgically induced neuronal injury and prevents POCD by activating the PI3K/Akt signalling pathway.

### 4.2 DEX activates the PGC-1α signalling pathway to protect against POCD

Peroxisome proliferator-activated receptor γ coactivator 1-alpha (PGC-1α) is a multifunctional protein that plays a critical role in various neurological disorders (Han et al., 2021). Overexpression of PGC-1α has been shown to significantly alleviate cognitive deficits (Han et al., 2020). High expression levels of PGC-1α have been observed in brain regions such as the cerebral cortex, striatum, and pallidum (Tritos et al., 2003). PGC-1α is a principal regulator of mitochondrial biogenesis and function. By improving mitochondrial performance, it reduces ROS levels, thereby mitigating hippocampal cell damage caused by chronic cerebral underperfusion and enhancing neuronal metabolic activity (Han et al., 2020). Amyloid β-protein (Aβ), a by-product of amyloid precursor protein (APP) processing, and neuronal loss constitute pathological hallmarks of AD (Senousy et al., 2022). Upregulation of PGC-1α inhibits Aβ pathology by modulating β-secretase activity, thus preventing Aβ production, reducing neuronal damage, and improving cognitive function in AD model mice (Motawi et al., 2022). Han et al. (2020) demonstrated that PGC-1α enhances synaptic plasticity, promotes energy metabolism in hippocampal neurons, and increases the expression of brain-derived neurotrophic factor (BDNF) and mitochondrial antioxidants, thereby alleviating cognitive dysfunction.

DEX significantly increases PGC-1α levels, thereby inhibiting mitochondrial damage and cellular inflammation. Li et al. (2018) demonstrated that DEX administration directly upregulates PGC-1α protein expression in traumatic brain injury (TBI)-affected regions, effectively reducing neuroinflammation, ROS production, neuronal degeneration, and apoptosis, while improving cognitive and behavioral outcomes. These findings suggest that DEX may serve as a potential therapeutic agent for POCD. In conclusion, PGC-1α plays a central role in the pathogenesis of neurological diseases by regulating mitochondrial function and ROS levels. DEX exerts neuroprotective effects against POCD by upregulating PGC-1α, offering a novel therapeutic target.

### 4.3 DEX activates the CREB/BDNF signalling pathway to protect against POCD

cAMP response element-binding protein (CREB) is a key regulator of neuronal growth and a critical molecular target for learning and memory processes (Carlezon et al., 2005; Barco et al., 2003). CREB-mediated gene expression, including that of BDNF, nerve growth factor (Fayaz et al., 2016), VGF (nerve growth factor inducible), and tissue plasminogen activator (t-PA), is essential for long-term memory formation and synaptic plasticity (Bourtchuladze et al., 1994; Won and Silva, 2008; Chen et al., 2012). Impairment of these signalling pathways is a potential mechanism underlying cognitive deficits in AD (Pláteník et al., 2014). Among these, BDNF, a direct target gene of CREB, plays a pivotal role in synaptic plasticity and memory formation (Lee and Silva, 2009; Cowansage et al., 2010; Miranda et al., 2019). Wang et al. (2020) demonstrated that CREB promotes BDNF gene expression and the anti-apoptotic protein Bcl-2. BDNF exerts neuroprotective effects by regulating hippocampal synaptic plasticity and promoting neurogenesis.

DEX has been shown to protect and restore neurological functions by activating CREB (Hu et al., 2017; Li et al., 2020). Taha et al. (2023) reported that DEX administration upregulated BDNF expression in the rat hippocampus, reducing oxidative stress, alleviating methotrexate (MTX)-induced neurotoxicity, and improving memory deficits. Additionally, Chen et al. (2024) demonstrated that DEX promotes hippocampal neurogenesis and reduces neuronal damage by activating the BDNF/CREB signalling pathway in neonates with hypoxic-ischemic brain damage (HIBD). This activation ameliorated neurological damage and cognitive dysfunction. In conclusion, DEX promotes neurogenesis and synaptic plasticity, attenuates neurotoxic effects, and improves cognitive function by activating CREB and its downstream target gene, BDNF.

### 4.4 DEX activates the nrf2/HO-1 signalling pathway to protect against POCD

Nuclear factor erythroid 2-related factor 2 (Nrf2) is a transcription factor that regulates genes involved in oxidative stress responses and drug detoxification. It plays a critical role in mitigating oxidative stress (Shao, 2022; Ngo and Duennwald, 2022). DEX has been shown to alleviate oxidative stress in various pathological conditions by activating the Nrf2 signalling cascade, which inhibits neuronal apoptosis and neurodegenerative processes associated with cerebral ischemia (Wang R. et al., 2023). However, there is a notable decline in Nrf2 activity has been observed in aged organisms (Zhang et al., 2015).

Heme oxygenase 1 (HO-1), a redox-sensitive enzyme, converts heme to biliverdin and exhibits both anti-inflammatory and antioxidant properties (Chen, 2014). Increased HO-1 expression with age is hypothesised to reduce toxic proteoglycan accumulation in AD, thereby ameliorating age-related cognitive decline (Hirose et al., 2003; Kurucz et al., 2018; Smith et al., 1994).

Nrf2 is a key regulator of HO-1 expression, promoting its upregulation (Syapin, 2008; Tonelli et al., 2018). Park et al. (2023) conducted a study on ischemic rats pre-treated with DEX, demonstrating elevated Nrf2 and HO-1 expression levels, alongside reduced caspase-3 activity. This suggests that DEX provides neuroprotective benefits through the Nrf2/HO-1 pathway. Furthermore, Li et al. (2019) demonstrated that DEX mitigates POCD after traumatic brain injury (TBI) by activating the Nrf2 pathway and upregulating downstream factors HO-1 and NQO-1, thereby reducing neuroinflammation-induced apoptosis. In summary, DEX exerts neuroprotective effects in various neurological disorders by activating the Nrf2/HO-1 signalling pathway, reducing neuronal apoptosis and inflammation, and providing protection against POCD.

### 4.5 DEX inhibits the TLR4/NF-κb signalling pathway to protect against POCD

Toll-like receptor 4 (TLR4) is a pattern recognition receptor within the toll-like receptor (TLR) family that plays a critical role in immune system functioning by recognising both pathogens and endogenous noxious stimuli. It induces innate and adaptive immune responses and is prominently expressed in the central nervous system, particularly in neural glial cells (Liu et al., 2021). TLR4 is involved in the regulation of nuclear factor kappa-B (NF-κB), a critical mediator of neuroinflammatory responses (Ghosh and Dass, 2016). The upregulation of TLR4 has been associated with deficits in memory and learning and is regarded as a significant contributor to the pathogenesis of POCD(Wang et al., 2013). NF-κB, a principal downstream pathway of TLR4, regulates the expression of inflammatory mediators, such as IL-1β, IL-6, and TNF-α. These cytokines promote cellular inflammation and apoptosis, contributing to cognitive impairments in POCD (Huang et al., 2019; Chi et al., 2015; Zhang D. et al., 2019). Under ischemic and hypoxic conditions, TLR4 activation initiates the NF-κB pathway, resulting in an inflammatory response that exacerbates neuronal apoptosis and neuropathic pain (He et al., 2020).

Recent studies have demonstrated that inhibiting the TLR4/NF-κB pathway constitutes a primary mechanism underlying the neuroprotective effects of DEX (Zhang et al., 2018). DEX modulates the TLR4/NF-κB pathway, thereby reducing levels of inflammatory cytokines such as TNF-α and IL-1β. Kim E. et al. (2017) reported that DEX inactivated the TLR4/NF-κB pathway, reducing inflammation and conferring neuroprotection against transient cerebral ischemia/reperfusion injury in rats. Similarly, Zhou X. Y. et al. (2019) found that DEX attenuated the through modulation of the TLR4/NF-κB pathway, significantly improving cognitive dysfunction in aged POCD mice. In conclusion, DEX exerts neuroprotective effects by inhibiting the TLR4/NF-κB pathway, thereby reducing inflammatory responses and mitigating POCD in elderly individuals.

### 4.6 DEX inhibits the JAK/STAT signalling pathway to protect against POCD

The Janus tyrosine kinase/signal transducer and activator of transcription (JAK/STAT) pathway consists of two protein families: JAK and STAT. In the central nervous system, the JAK/STAT pathway is primarily involved in processes such as hormone release, inflammation, tumour formation, and gene regulation during development (Nicolas et al., 2013). Specifically, the JAK2/STAT3 signalling pathway has been implicated in the progression of cerebral ischemia-reperfusion injury (CIRI) (Zhong et al., 2021). CIRI upregulates the expression of phosphorylated JAK2 (p-JAK2) and phosphorylated STAT3 (p-STAT3) and induces the release of various inflammatory mediators. In contrast, inhibitors of JAK2, such as AG490, and STAT3 inhibitors exhibit significant neuroprotective effects, suggesting potential therapeutic value in the treatment of neurodegenerative diseases (Satriotomo et al., 2006).

Similar to JAK/STAT inhibitors, DEX suppresses the activation of JAK2 and STAT3 in the cerebral cortex, thereby attenuating neuroinflammation and apoptosis (Liu et al., 2022). Chen et al. (2017) reported that DEX reduced neuronal damage in the hippocampus of rats undergoing cardiopulmonary bypass (CPB). Their findings indicated that CPB led to increased levels of p-JAK2 and p-STAT3 proteins, while DEX ameliorated POCD by inhibiting the JAK2/STAT3 pathway. In contrast, (Kim H. C. et al. (2017) observed that in aged mice pre-treated with DEX before exposure to isoflurane, DEX attenuated isoflurane-induced cognitive deficits despite elevated phosphorylation levels of JAK2 and STAT3. This discrepancy may stem from differences in experimental models, subjects, conditions, and the degree of injury. Chen et al. used a rat model of CPB, whereas Kim et al. employed an aged mouse model exposed to isoflurane, involving distinct pathophysiological mechanisms. Additionally, differences in the age of the animals and the severity of injuries (e.g., CPB versus isoflurane exposure) may account for variations in the effects of DEX. These findings suggest that the neuroprotective impact of DEX on the JAK2/STAT3 pathway is influenced by specific experimental variables.

### 4.7 DEX inhibits the Drp1-Bax signalling pathway to protect against POCD

Dynamin-related protein 1 (Drp1) is a member of the GTPase family and plays a critical role in regulating mitochondrial morphology, distribution, and remodelling, as well as neuronal injury and synaptic degeneration (Kim et al., 2018; Manczak et al., 2011). Activation of Drp1 has been shown to promote apoptosis by facilitating the mitochondrial translocation of Bcl2-associated X (Bax), increasing cytochrome C release, and activating the caspase-3/-9 signalling pathway. Additionally, Drp1 mediates metabolic disturbances and depletes mitochondrial glutathione levels, impairing free radical scavenging capacity. These effects increase mitochondrial ROS production, exacerbating mitochondrial dysfunction and forming a pathological basis for POCD (Duan et al., 2020).

**FIGURE 1:**
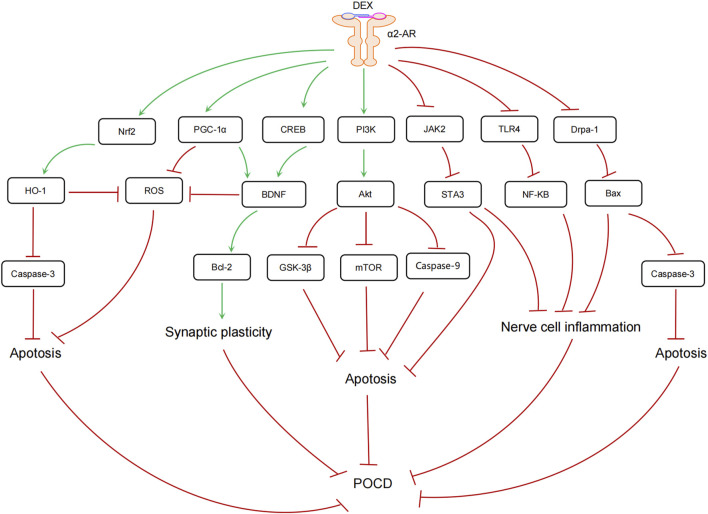
Role of Multiple Signalling Pathways in POCD. DEX has been demonstrated to bind to α2-AR, thereby promoting synaptic plasticity, inhibiting oxidative stress, suppressing neuronal inflammation and apoptosis, and ultimately inhibiting POCD through the activation of multiple signalling pathways. The following pathways are involved: Nrf2/HO-1, PGC-1α, CREB/BDNF, PI3K/Akt, JAK/STAT, TLR4/NF-κB, and Drp1-Bax.

Studies have shown that Drp1 exacerbates isoflurane-induced cognitive impairments in rats (Zhang et al., 2014). Shan et al. (2018) reported that sevoflurane anaesthesia significantly increased the expression of Drp1 and Bax, both of which promote neuronal apoptosis, thereby impairing learning and memory. However, DEX inhibited these increases, mitigating neurological damage. Similarly, Qian et al. (2015) observed that splenectomy under general anaesthesia in aged mice caused severe cognitive impairments. Preoperative administration of DEX reduced hippocampal levels of pro-inflammatory factors TNF-α and IL-1β and downregulated apoptosis-related factors, including caspase-3 and Bax, thereby preventing the onset of POCD. These findings suggest that the activation of Drp1 and Bax contributes to apoptosis and mitochondrial dysfunction, exacerbating cognitive impairments. Conversely, DEX ameliorates neurological damage and prevents POCD by inhibiting Drp1 and associated apoptotic factors (Figure 1).

## 5 Discussion and summary

DEX, a novel α2-adrenoceptor agonist, shows great potential as a therapeutic agent for mitigating POCD in elderly patients. In a network meta-analysis comparing different anaesthetic drugs regarding the incidence of POCD in the elderly, Zeng et al. (2023) demonstrated that DEX (12.9%) and sufentanil (6.3%) were the most effective drugs for reducing the incidence of POCD in the elderly. In particular, DEX significantly reduced the incidence of POCD compared to placebo (27.7%) and sevoflurane (24.0%). DEX has been demonstrated to exert neuroprotective effects, which may be attributed to its ability to reduce the expression of pro-inflammatory factors by activating relevant signalling pathways, while simultaneously inhibiting the stress response and apoptosis, thereby reducing neuronal toxicity and ameliorating the occurrence of POCD by facilitating synapse formation and providing neurotrophic nutrition (Zhou M. et al., 2019). In clinical practice, a 15-min intravenous infusion of DEX at a loading dose of 0.5 μg/kg is typically administered 15 min prior to the induction of anaesthesia, and then maintained at a continuous rate of 0.5 μg/kg/h until the conclusion of the procedure (Lu et al., 2017). However, due to inter-patient variability, the dose, duration, and application of DEX may vary. It is noteworthy that DEX has been observed to predispose to the development of adverse effects, including hypotension and bradycardia, in the perioperative period. These side effects may be multifactorial and dose-dependent (Nguyen et al., 2017). Low doses of DEX have been observed to induce a decrease in central sympathetic excitability and a reduction in norepinephrine release. Conversely, high doses of DEX have been linked to the development of transient hypertension with reflex bradycardia (Nguyen et al., 2017). It is therefore recommended that further in-depth studies with larger sample sizes and a greater understanding of the mechanism of action of DEX should be conducted in the future, in order to ensure the optimal clinical application of DEX.

The occurrence of POCD is associated with age and anaesthetic drugs, but also other risk factors, including low level of education, type and duration of surgery, and postoperative pain (Zeng et al., 2023). It has been demonstrated that the brain networks of individuals with low cognitive reserve demonstrate reduced resilience to the damage caused by reduced flexibility and efficiency when compared to those with high cognitive reserve (Foubert-Samier et al., 2012). For instance, patients with limited educational attainment may demonstrate heightened Aβ accumulation and augmented tau deposition during surgical procedures, thereby intensifying the incidence of POCD (Vemuri et al., 2015). Furthermore, the reduction in tissue trauma that is associated with minimally invasive surgery results in a less severe postoperative inflammatory response. Consequently, the incidence and severity of POCD are diminished (Bhushan et al., 2021). Furthermore, reducing the duration of surgery not only mitigates the release of pro-inflammatory mediators but also minimises the necessity for sedative and analgesic medications, which is pivotal for the prevention of POCD (Olotu, 2020). In addition, severe postoperative pain may also precipitate postoperative delirium, which in turn may lead to the development of POCD (Leung et al., 2013). The APOE4 genotype is strongly linked to the development of Alzheimer’s disease. Individuals with this genotype have been shown to have a markedly elevated risk of developing POCD within 3 months of surgery (Bertram and Tanzi, 2008). However, the precise mechanism by which this occurs remains unclear.

In conclusion, DEX has been demonstrated to be an effective intervention for the prevention of POCD in elderly patients. Further investigations are required to elucidate the precise mechanisms by which DEX modulates the nervous system and to determine the optimal dosage and timing for its application in surgical anaesthesia for this population. Additionally, exploring the combination of DEX with other pharmacological agents may enhance its efficacy in managing POCD.
